# The curative-to-preventive perspective shift of medical students through community dementia programs: a qualitative study based on transformative learning theory

**DOI:** 10.3389/fpubh.2025.1708455

**Published:** 2026-01-12

**Authors:** Ruiyu Huang, Zhu Zhu, Mei He, Pan Cai, Rulai Wang, Linfang Ran, Xiaofang Yang, Baolu Zhang

**Affiliations:** 1 School of Continuing Education, Guiyang Healthcare Vocational University, Guiyang, China; 2 The Second Affiliated Hospital of Zunyi Medical University, Zunyi, China; 3 The First People’s Hospital of Zunyi City, Zunyi, China; 4 The Third Affiliated Hospital of Zunyi Medical University, Zunyi, China; 5 School of Nursing, Guiyang Healthcare Vocational University, Guiyang, China; 6 Guiyang Maternal and Child Health Care Hospital, Guiyang, China; 7 School of Nursing, Southwest Medical University, Luzhou, China

**Keywords:** preventive healthcare, community dementia prevention, medical students, transformative learning theory, qualitative study

## Abstract

**Background:**

Preventive healthcare has become a global health priority, yet a significant implementation gap persists in community settings. Traditional medical education predominantly emphasizes curative approaches, inadequately preparing students for the growing demands of community-based preventive care. With the rising prevalence of dementia, a condition where early intervention and prevention strategies are crucial, there is an urgent need to shift medical students’ perspectives from treatment-focused to prevention-oriented practice. This study aimed to explore how medical students’ participation in community dementia prevention programs facilitated their curative-to-preventive perspective shift, using transformative learning theory (TLT) as the conceptual framework.

**Methods:**

A qualitative descriptive design was employed, conducting semi-structured interviews with 21 medical students from community dementia prevention programs at a medical university in Guizhou Province, China. Data analysis utilized Braun and Clarke’s deductive thematic analysis framework guided by TLT.

**Results:**

The study identified four major themes and 11 sub-themes. The themes included: (1) Cognitive awakening and reflection, encompassing encountering disorienting dilemmas, self-examination and critical reflection, and role perception transformation; (2) Skill acquisition, including building preventive knowledge frameworks, community intervention design, and development of practical skills; (3) Practice integration and role reconstruction, comprising health promoter role practice, prevention practice capabilities enhancement, and professional identity evolution in prevention; (4) Personal experience-driven preventive healthcare awakening, including impact of family dementia experience and experience-triggered preventive awareness.

**Conclusion:**

This qualitative study identified four themes characterizing medical students’ transformative learning (TL) journey in community dementia prevention programs. The synergistic interaction between structured TLT-based progression and experiential pathways facilitated students’ paradigm shift from treatment-centered to prevention-focused healthcare approaches. Our findings recommend that medical educators should intentionally design learning environments that incorporate TLT, integrate community-based practice into core curricula through problem-driven approaches, and systematically leverage students’ personal experiences to foster critical reflection and enhance learning motivation, thereby preparing future healthcare professionals to serve as prevention-oriented change agents.

## 1 Introduction

Preventive healthcare has emerged as a global health priority, alongside the increasing recognition that community-based interventions can significantly reduce disease incidence, decrease healthcare expenditures, and improve population health outcomes (1, 2). Despite widespread acknowledgment of prevention’s importance, a significant implementation gap persists in community settings. These gaps are particularly evident in the insufficient human resources among community healthcare professionals, lack of specialized training, and limited public awareness of preventive healthcare (3, 4).

Among various preventive healthcare challenges, dementia prevention represents one of the most urgent issues (5). Statistics has indicated that the global prevalence of dementia is rapidly increasing, with projection estimating 152 million cases by 2050 (6). More critically, with no effective curative treatments currently available, dementia potentially results in complete loss of self-care capacity among older adults (7), thereby generating enormous care burden and economic burden (8). Therefore, early prevention of dementia becomes crucial. Community-based interventions serve as key approaches for dementia prevention in the early phase. Previous systematic reviews and meta-analyses have demonstrated that community-level early prevention interventions have tremendous potential to reduce dementia incidence, minimize healthcare expenditures, and alleviate family caregiving burden (9). However, previous research revealed that despite substantial demand for dementia prevention services, most primary care centers lacked accessible preventive services due to severe shortages of dementia prevention providers in community settings, limited public awareness of prevention strategies, and insufficient healthcare system support for preventive care programs (10, 11). In China, general practitioners have faced significant barriers to delivering early screening and preventive interventions regarding dementia. These challenges stem from inadequate institutional support, limited access to specialized training programs, and extensive existing workloads that limit dementia preventive care delivery (12–14).

Medical education, as a key component in training future healthcare professionals, faces the challenge of how to effectively cultivate students’ preventive healthcare capabilities. The traditional medical education models emphasize disease diagnosis and treatment while providing limited preventive healthcare educational content (15). This educational approach proves insufficient to prepare future healthcare professionals for the growing community preventive healthcare needs. In response to rapid change in healthcare, medical schools globally are continuously incorporating public health curricula into their existing programs, seeking to strengthen future healthcare professionals’ capacity for preventive care and better serve evolving community health demands (16, 17). Notably, it is recommended to provide experiential learning experiences for medical students to shift their existed perception and tackle real-world complex challenges (18). Earlier studies have revealed the progressive integration of community-based experiential learning into medical curricula. For instance, Gao et al. described the evolution of a community-centered learning module that shifted from traditional medical school-driven designs toward substantial involvement of a community-based organization in curriculum development and implementation (19). Similarly, Anselin et al. reported the implementation of a longitudinal experiential curriculum for all second-year medical students, designed to address gaps in social determinants of health education through structured community partnerships (20). Yet limited research has explored how these community-engaged learning experiences specifically influence medical students’ perceptions of preventive healthcare. In the context of dementia education, existing research has primarily focused on improving medical students’ attitudes and clinical competencies through dementia patient-centered approaches (21). Existing research indicated that medical students generally demonstrated insufficient knowledge about dementia, suggesting that educational interventions introducing students to dementia patients could enhance their knowledge, skills, and attitudes while reducing stigmatization toward dementia (22). However, current dementia-related medical education research has predominantly concentrated on clinical care delivery and attitude formation, with insufficient focus on the transformative learning (TL) processes that guide students toward preventive care paradigm.

To better understand the mechanisms underlying how community-engaged experiential learning influences medical students’ perceptions of preventive healthcare, Mezirow’s transformative learning theory (TLT) provides a valuable theoretical framework for examining cognitive shifts among community-based experiences (23). TLT has been widely applied and validated across various fields, proving effective in explaining how learners critically evaluate existing beliefs, develop professional competencies, and incorporate new perspectives into medical practice (24). Prior research explored the application value of TLT in understanding how clinical experiences shape professional identity development (25). However, no research has applied the TLT framework to examine how medical students develop their perceptions of preventive healthcare. Therefore, this study addresses this gap by investigating how participation in community dementia programs transforms medical students’ perspectives from curative-focused training toward preventive healthcare practice. By applying TLT as a theoretical framework, we aim to identify specific learning mechanisms that facilitate curative-to-preventive perspective shift among medical students.

## 2 Methods

### 2.1 Research design

This study employed a qualitative descriptive design utilizing semi-structured interviews to explore the TL experiences of medical students participating in community dementia scheme. Qualitative research methods enabled exploration of how medical students reconstructed their professional identity and developed preventive healthcare competencies. These processes involved subjective experiences and psychological transformations that could not be adequately measured through quantitative methods alone. Meanwhile, semi-structured interviews were chosen as the primary data collection method because they provided the necessary flexibility for in-depth exploration of students’ cognitive transformation, skill development, and identity reconstruction processes. Additionally, it allowed participants to describe their unique transformative experiences in community-based preventive healthcare activities in their own words. This study strictly adhered to the Consolidated Criteria for Reporting Qualitative Research (COREQ) checklist (26), thereby enhancing methodological integrity and reporting transparency.

### 2.2 TLT

This research was guided by Mezirow’s TLT, which propose that TL occur through a series of phases including critical reflection on assumptions, exploration of new roles and relationships, and integration of new perspectives into one’s life. Mezirow’s TLT presents a sequential ten-phase process that systematically guides perspective transformation (27, 28). The framework begins with a disorienting dilemma (Phase 1) that disrupts existing assumptions, prompting self-examination accompanied by guilt or shame (Phase 2) and critical assessment of existing assumptions (Phase 3). Learners subsequently recognize their experience as shared among others facing similar transformations (Phase 4), then explore new roles, relationships, and actions (Phase 5) while developing concrete implementation plans (Phase 6). Following this planning stage, the process advances through knowledge and skill acquisition (Phase 7), which then enables temporary testing of new roles (Phase 8), and systematic development of competence and confidence within these emerging roles (Phase 9), ultimately culminating in the reintegration of transformed perspectives into one’s life context (Phase 10). This sequential progression demonstrates the systematic nature of TL, emphasizing both cognitive restructuring and practical application as integral components of learning transformation. This sequential progression demonstrates the systematic nature of TL, making it particularly suitable for analyzing how medical students undergo perspective transformation from curative to preventive healthcare approaches through community-based preventive healthcare engagement. Building upon this theoretical foundation, we designed interview guides structured around the 10 phases and systematically utilized the TLT framework to identify and extract themes related to TL experiences during data analysis.

### 2.3 Setting and procedures

This study was conducted at a medical university in Guizhou Province, one of the southwestern provinces of China. Participant recruitment and interviews were conducted from February 2024 to May 2024. According to China’s Seventh National Population Census Guizhou Province has 11.56% of its permanent residents aged 65 and above, with education levels below the national average (29), which are important risk factors for increased dementia prevalence. In response to the limited dementia prevention services at community-level healthcare institutions, a student organization at the medical university initiated a comprehensive community dementia undertaking. The community dementia programs, specifically focused on dementia prevention, was established by undergraduate medical students (including first-, second-, and third-year students) as a volunteer-driven initiative. This program was implemented across multiple community venues, including senior centers, primary healthcare facilities, and residential care homes. Medical students served as trained volunteers who conducted cognitive function assessments for older population and delivered multi-domain interventions designed to enhance cognitive function in older adults. To ensure program quality, all training sessions and practical implementations were supervised and guided by university faculty and clinical specialists from the Guizhou Association for Alzheimer’s disease. Student volunteers participated on a voluntary basis during weekends and weekday evening hours.

### 2.4 Participants

Purposive sampling was employed to recruit participants representing different levels of programs involvement. The inclusion criteria were: (i) second- and third-year students who participated in the community dementia programs in 2024; (ii) voluntary participation in the research with signed written informed consent; and (iii) demonstrated ability for self-reflection and articulate expression. Students were excluded from the study if they met either of the following criteria: (i) participated in fewer than four sessions of the dementia prevention programs or (ii) first-year students whose involvement was limited to preliminary training and observational activities, with minimal hands-on experience in dementia prevention volunteer work. Before the interviews, researchers contacted the programs leader, who preliminarily inquired about volunteers’ willingness to participate in the study and provided their phone numbers. All 30 students participating in the 2024 community dementia programs received telephone invitations from researchers who were independent of the prevention programs. Nine students were excluded for not meeting the inclusion criteria, resulting in a final sample size of *n* = 21. Participants were all second-and third-year medical students who had completed dementia prevention training and were capable of independently conducting community cognitive intervention activities.

### 2.5 Data collection

Data collection was conducted by the research team consisting of three medical students with extensive knowledge of dementia-related topics (MH, RLW, and LFR) and five members with specialized expertise in geriatric medicine and knowledge of dementia care (RYH, BLZ, ZZ, PC, and XFY). To minimize bias, interviews were conducted by medical students who were not involved in the programs’ implementation. Participants were recruited by an independent team member via telephone, who explained the research objectives, procedures, and time requirements. The interview guide was developed based on Mezirow’s TLT, focusing on three key domains: experiences with community dementia programs, shifts in preventive healthcare perspectives, and professional role development. The interview questions included: (i) What motivated your participation in the community dementia program? (ii) What challenges or difficulties have you encountered in the dementia prevention program? (iii) Which specific experiences prompted you to reconsider your understanding of preventive healthcare? (iv) What changes and gains do you feel you have experienced through this program? (v) How do you think this experience will influence your future choices and developmental direction?

Interviews were conducted in private conference rooms at the medical university, lasting 30–60 min each. Each interview session included one participant and two interviewers. All sessions were audio-recorded with informed consent. Data collection was monitored continuously for thematic saturation. After interviewing all 21 eligible participants, we confirmed that thematic saturation had been achieved, as the final interviews yielded no substantial new themes or insights beyond those identified in earlier analyses. All interviews were transcribed verbatim and cross-checked against the original recordings to ensure transcription accuracy. Subsequently, transcripts were returned to all participants for member checking to verify accuracy and completeness. Face-to-face meetings were arranged with each participant within 2 weeks of their interview to review their transcripts. All participants confirmed that the transcripts accurately captured their statements, with no discrepancies identified or corrections requested. After completing the thematic analysis, a summary of key findings was shared with participants through individual face-to-face sessions to ensure our interpretations aligned with their intended meanings. All participants confirmed that the themes accurately reflected their experiences, with no requests for modifications to the analytical framework. Field notes were maintained throughout the data collection process to capture contextual information, document researchers’ observations, and record non-verbal cues during in-depth interviews. Specifically, field notes informed our understanding of participants’ emotional reactions, behavioral cues, and the contextual nuances that enriched thematic interpretation. Furthermore, interviewers had no evaluative relationship with participants. All participants were explicitly informed that their responses would not affect their academic standing and were encouraged to share authentic experiences.

### 2.6 Data analysis

This study utilized Braun and Clarke’s deductive thematic analysis approach, which was systematically combined with the TLT (30). After data collection was completed, the researchers responsible for interviews (RYH, MH, RLW, LFR) transcribed all interview recordings verbatim and reviewed them repeatedly, while generating preliminary observation notes. During this initial stage, researchers adopted an open observational stance, processing data without preset theoretical assumptions and focusing on participants’ authentic expressions to ensure comprehensive capture of their genuine experiences. To control for researcher bias, all researchers declared no conflicts of interest and regularly discussed potential bias issues in team meetings. Subsequently, three researchers (BLZ, ZZ, and XFY) independently conducted systematic line-by-line coding of the records. The researchers then compared their respective initial coding, identifying areas of convergence while developing and continuously refining the coding scheme. Disagreements encountered during the coding process were resolved through consultation with a third researcher (PC), and researcher triangulation was implemented to enhance the credibility and reliability of the analysis (31). Furthermore, the analytical approach integrated deductive and inductive strategies to ensure comprehensive data exploration. This study employed a deductive analytical approach guided by TLT. This theory-driven approach enabled systematic identification and extraction of themes directly related to Mezirow’s concept of TL experiences. Subsequently, inductive thematic analysis was employed to identify emerging themes beyond the original theoretical framework, revealing other factors and processes that influence medical students’ learning experiences but are not explicitly mentioned in TLT. This analysis integrated theory-driven themes and emerging themes while maintaining close connection with the original data to ensure authentic representation of participants’ TL experiences in the community dementia scheme.

### 2.7 Ethical considerations

Ethical approval was obtained from Guiyang Healthcare Vocational University in China (IRB number: GKD202312). All participants provided written informed consent before participation. Confidentiality was maintained by using participant codes rather than names in transcripts and reports. Interview recordings and transcripts were stored securely with access limited to the research team.

## 3 Results

In 2024, a total of 21 medical undergraduate students who participated in the community dementia prevention programs agreed to participate and were interviewed. The participants included 13 female students (61.9%) and 8 male students (38.1%), aged between 18 and 21 years. The sample covered different academic years:13 s-year students (61.9%) and 8 third-year students (38.1%). Among them, all 21 students were able to independently conduct community prevention activities as volunteers. Table 1 provides an overview of the characteristics of the participants.

**Table 1:** Participant characteristics.

Codes	Gender	Ages	Major	Grade
P1	Female	20	Nursing	Second-year
P2	Female	21	Rehabilitation Therapy	Third-year
P3	Male	20	Nursing	Second-year
P4	Female	20	Public Health Management	Second-year
P5	Male	21	Nursing	Third-year
P6	Female	20	Nursing	Third-year
P7	Female	20	Public Health Management	Second-year
P8	Male	19	Nursing	Second-year
P9	Male	21	Public Health Management	Third-year
P10	Female	20	Nursing	Second-year
P11	Male	19	Rehabilitation Therapy	Second-year
P12	Female	21	Rehabilitation Therapy	Second-year
P13	Female	21	Smart Health and Older Population Management	Third-year
P14	Female	20	Rehabilitation Therapy	Second-year
P15	Male	19	Nursing	Second-year
P16	Female	21	Public Health Management	Second-year
P17	Female	21	Nursing	Third-year
P18	Male	20	Nursing	Second-year
P19	Female	21	Clinical Medicine	Second-year
P20	Male	20	Smart Health and Older Population Management	Third-year
P21	Female	20	Nursing	Second-year

**Figure 1:**
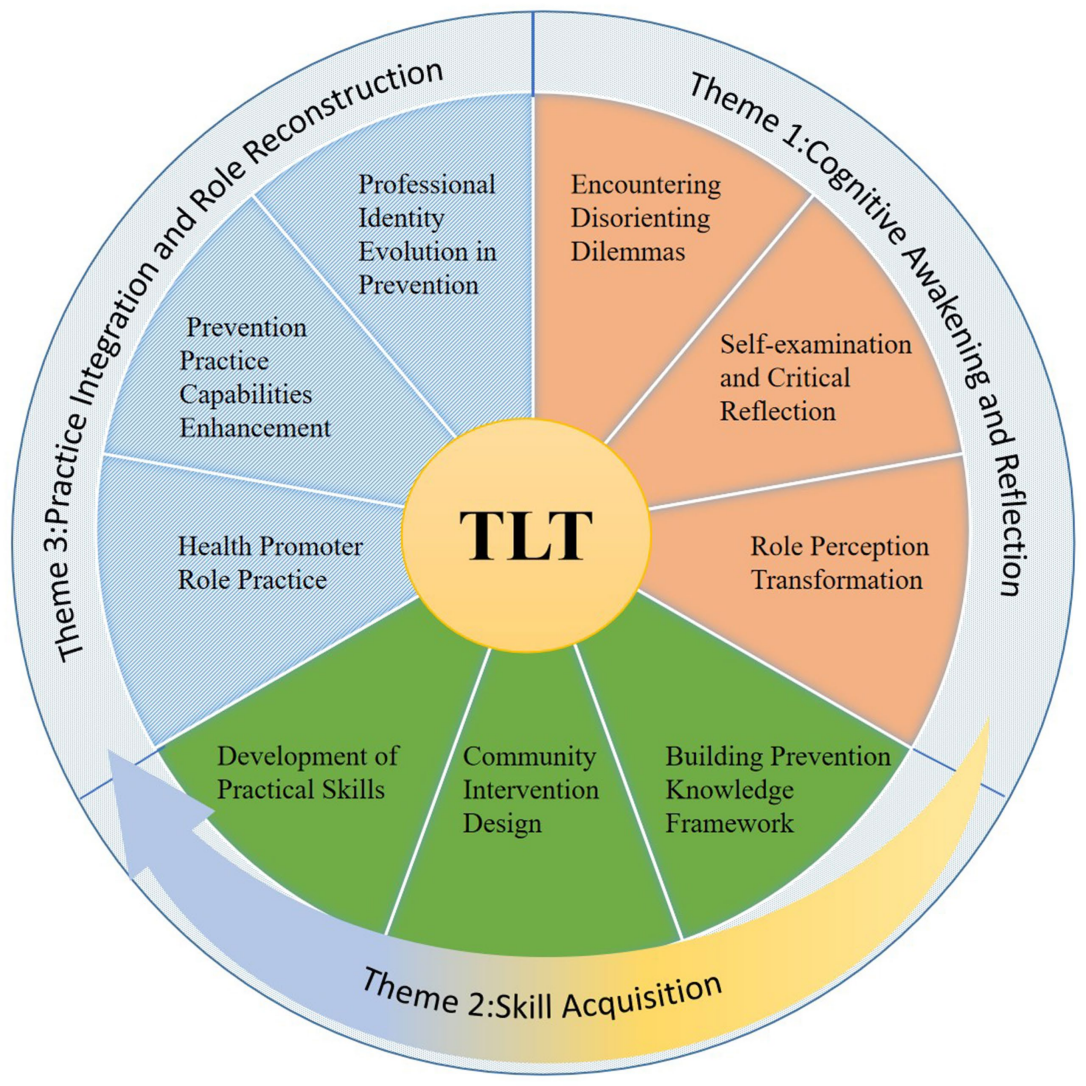
Themes, sub-themes, and categories derived from the TLT.

Through analysis of participant interviews, our research identified three themes corresponding to 10 stages of TLT, encompassing nine sub-themes. These themes represent the dynamic progression of TL experienced by medical students when participating in community dementia prevention plans. These three themes are: (i) Cognitive Awakening and Reflection, (ii) Skill Acquisition, and (iii) Practice Integration and Role Reconstruction. Table 2 presents a comprehensive overview of the themes, sub-themes, and categories. Figure 1 illustrates the relationship between these themes, sub-themes, and categories within the framework of TLT.

**Table 2:** The overview of themes, sub-themes, and categories.

TLT stage	Themes	Sub-themes	Categories
1, 2, 3, 4	Cognitive awakening and reflection	Encountering disorienting dilemmas	Gap between theoretical knowledge and community healthcare needs
			Curriculum gaps in preventive healthcare education
			Recognition of current medical education limitations
		Self-examination and critical reflection	Prevention knowledge deficits
			Insufficient basic knowledge
			Lack of practical application knowledge
		Role perception transformation	Identity shift from healer to prevention advocate
			Recognition of preventive healthcare values
5, 6, 7	Skill acquisition	Building prevention knowledge framework	Learning to raise awareness
			Identifying optimal prevention timing
			Mastering approaches to prevent dementia
		Community intervention design	Intervention design considering community conditions
			Converting prevention theories into practical approaches
			Tailored prevention plan development skills
		Development of practical skills	Professional terminology conversion
			Mastering health education methods
			Learning to assess community residents’ needs
			Developing patience and empathy with older adults
8, 9, 10	Practice integration and role reconstruction	Health promoter role practice	Active dissemination of dementia prevention knowledge
			Capable of independently conducting dementia early screening activities
			Learning to guide residents in early cognitive intervention
		Prevention practice capabilities enhancement	Skillfully identify risk factors for dementia
			More confident in answering residents’ consultations about dementia prevention
			Increasingly confident in developing personalized dementia prevention plans
		Professional identity evolution in prevention	Recognition of prevention and treatment as integrated
			Recognizing disease prevention as an important responsibility of healthcare providers
			Family chronic disease prevention practice
			Career aspirations in preventive healthcare
Not applicable TLT stage	Personal experience-driven preventive healthcare awakening	Impact of family Dementia experience	Witnessing the suffering caused by family members’ cognitive decline
			Experiencing the heavy burden of family caregiving
		Experience-triggered prevention awareness	Active exploration of prevention knowledge
			Reorientation toward prevention-focused medical practice

In presenting our findings, we included the full range of selected codes from the complete coding framework (Supplementary Table 1) and added Supplementary Table 2 with illustrative quotes organized by themes and sub-themes.

### 3.1 Cognitive awakening and reflection (stages 1–4)

The first theme, cognitive awakening and reflection, corresponds to stages 1–4 of TL. This theme encompasses three subthemes that align with Mezirow’s initial phases: “encountering disorienting dilemmas” corresponds to Phase 1, where participants described moments when their hospital-centric curative assumptions were challenged by community realities; “self-examination and critical reflection” aligns with Phases 2–3, where participants reported experiencing feelings of inadequacy and questioning their previous understanding of medical practice; and “role perception transformation” corresponds to Phase 4, where participants described recognizing that their peers shared similar transformative experiences and began to acknowledge prevention as part of medical professionalism.

#### 3.1.1 Encountering disorienting dilemmas

Many medical students report a gap between theoretical medical knowledge and community healthcare needs. Participants identified obvious curriculum deficiencies in preventive care within traditional medical education and became aware of its limitations. When students realize the disconnection between classroom learning and actual preventive healthcare needs, this cognitive conflict prompts them to rethink the essence of medical practice.

*“Our curriculum primarily focused on the pathogenesis, symptoms, and care of dementia, with little mention of prevention strategies. But when I began working in the field, I realized what really needed to happen was to help seniors develop preventative awareness and guide them toward a healthier lifestyle.” (P6)*

*“When conducting dementia prevention activities in the community, I realized that we needed to consider not only the accuracy of medical content but also how to enhance healthy behaviors, address cultural differences, and address the various concerns and worries of seniors about dementia prevention. These practical skills are almost completely missing from our current medical education.” (P4)*

*“I used to think that medical professionals’ responsibilities were solely to treat patients. But when I saw that the mental health of the older population in our community was significantly better than that of those already suffering from dementia, I suddenly realized: Wouldn't it be more meaningful to help them maintain this state of mind?” (P11)*

#### 3.1.2 Self-examination and critical reflection

After experiencing difficulties, medical students engaged in profound self-reflection and began to critically examine their knowledge base and understanding of their professional capabilities. They realized their limited knowledge of preventive healthcare, identified blind spots in basic knowledge, recognized their inability to combine theory with the actual situation of older population in the community, and acknowledged their lack of practical application of knowledge. The students expressed concerns about their insufficient professional preparation and recognized the need to bridge the significant gap between theoretical learning and practical application.

*“Previously, I knew almost nothing about dementia prevention and had no understanding of how to prevent it or control risk factors. After the training, I realized that many daily behaviors could serve a preventive role.” (P2)*

*“The dementia screening made me realize how little I knew. Although I had learned about MMSE and MoCA in class, I felt overwhelmed when applying them in practice. This made me realize the huge gap between rote memorization and actual application.” (P5)*

*“I realized there was a huge gap between textbook knowledge and actual practice. We had learned multidisciplinary prevention theories, but I didn't know how to apply these concepts to the specific situations of older population. Because each person's situation was different, it was difficult for me to develop practical plans that truly suited them.” (P16)*

#### 3.1.3 Role perception transformation

As students progressed through critical reflection, they began to reimagine their professional identity and the scope of medical practice. Students gradually recognized that healthcare providers’ responsibilities should not be limited to disease treatment but should extend to broader responsibilities for health promotion and disease prevention, while gaining a new understanding of the importance of preventive work.

*“Before participating in this dementia prevention plans, I thought healthcare meant treating patients in hospitals. But working with community members made me realize that we can actually help prevent diseases from occurring in the first place.” (P1)*

*“I used to think that prevention was not as important as treatment. But through my involvement in the prevention programs, I realized that preventing cognitive decline is more valuable.” (P9)*

### 3.2 Skill acquisition (stages 5–7)

This theme corresponds to stages 5–7 of Mezirow’s theory. The three subthemes align with these phases: “building prevention knowledge framework” corresponds to Phase 5, where participants described exploring new preventive healthcare roles and actively seeking knowledge beyond curative medicine; “community intervention design” aligns with Phase 6, where participants reported planning concrete action strategies for community dementia prevention; and “development of practical skills” corresponds to Phase 7, where participants described acquiring specific competencies including health education delivery, cognitive decline assessment, and community engagement techniques.

#### 3.2.1 Building prevention knowledge framework

After recognizing their knowledge deficiencies, students began systematic learning of prevention-related knowledge, learning how to effectively raise public awareness about prevention. Students conducted in-depth studies on how to identify and grasp the critical timing for disease prevention, particularly understanding the “golden period” concept in dementia prevention, while mastering various evidence-based dementia prevention methods and strategies.

*“I have found that older population have misconceptions about dementia and lack basic prevention knowledge. Therefore, raising awareness of prevention among the older population is crucial. Only by helping them understand the importance of prevention can they truly participate in the program.” (P20)*

*“During the community screening process, I discovered that many older population ignore slight memory changes, considering them normal signs of aging. However, when we emphasize the importance of early screening and inform them that intervention during the mild cognitive decline stage helps maintain better quality of life, they all actively participate.” (P12)*

*“Through training, I learned that risk factors for cognitive impairment include hypertension, diabetes, hyperlipidemia, and others. During health education, we should teach older population to control these chronic diseases through diet, exercise, medication, and other methods to reduce the risk of developing the condition.” (P3)*

#### 3.2.2 Community intervention design

Medical students learned to transform abstract prevention theories into concrete intervention programs suited to community conditions, demonstrating proactive practical capabilities. They were able to thoroughly analyze different communities’ resource conditions and cultural backgrounds to design locally appropriate prevention activity programs, reflecting an important shift from standardized medical services to personalized community health services.

*“Considering the community’s resource constraints, we used playing cards and household items to design simple memory games, integrating cognitive training with daily activities. This approach provided effective dementia prevention interventions without requiring expensive resources.” (P21)*

*“We learned to simplify professional intervention methods into forms that older population could easily accept. For example, when using the MMSE scale, we changed ‘100 minus 7’ to ‘If you have 100 yuan and spend 7 yuan on groceries, how much money do you have left?’ This approach was more closely related to their daily life experiences.” (P13)*

*“Each older population situation was different-some preferred physical activities while others preferred sedentary activities, and their educational levels also varied. We had to design personalized prevention plans based on each individual’s characteristics, which tested our professional competency and innovative thinking.” (P8)*

#### 3.2.3 Development of practical skills

Students developed the multifaceted capabilities needed to effectively conduct community prevention work. They could transform profound medical theories into easily understandable health knowledge, mastered diverse educational methods and techniques, and could effectively assess community residents’ health needs and cultural backgrounds. Students also demonstrated sufficient patience and warm empathy when communicating with older population, becoming bridges connecting professional knowledge with community health.

*“Seeing their bewildered expressions, I knew my explanation had exceeded their comprehension. I would immediately change my approach, using familiar life scenarios to help them understand. For example, when explaining ‘executive function’, ‘I would say,’ It’s like cooking-you need to remember the sequence of washing vegetables, cutting vegetables, and cooking vegetables.” (P10)*

*“I realized that the foundation of health education lies in changing behavior, not merely transmitting information. Therefore, we focus on helping them find their own motivation and practical methods for change. For example, when promoting executive function for dementia prevention, we share the power of daily puzzles and help participants set achievable goals.” (P14)*

*“Through field research experience, I discovered that different communities and population groups have vastly different needs. Older population in communities with higher education levels tend to place greater emphasis on scientific evidence, while older population living alone require additional emotional support and social connections. This made me understand the importance of needs assessment.” (P18)*

*“Through long-term contact, I began to understand the psychological state of older population. They feel anxious about the decline in their physical functions and confused about the transformation of their social roles. After understanding them, I became more empathetic in communication and was able to think from their perspective.” (P19)*

### 3.3 Practice integration and role reconstruction (stages 8–10)

This theme corresponds to stages 8–10 of Mezirow’s theory. The three subthemes align with these phases: “health promoter role practice” corresponds to Phase 8, where participants described trying out new preventive healthcare roles in community settings; “prevention practice capabilities enhancement” aligns with Phase 9, where participants reported building competence and confidence through repeated practice; and “professional identity evolution in prevention” corresponds to Phase 10, where participants described integrating prevention-oriented values into their understanding of physician roles and career aspirations.

#### 3.3.1 Health promoter role practice

Students actively embraced and demonstrated capabilities in new roles as health promoters and prevention advocates, proactively disseminating dementia prevention knowledge in communities. They could independently conduct community screening activities and guide older population in identifying early signs of dementia while providing hands-on instruction in early cognitive intervention techniques. Students demonstrated professional service capabilities and strong community engagement skills in practice, successfully achieving role transformation from medical theory learners to community health service providers, reflecting effective integration of medical education with community needs.

*“Through the cognitive impairment prevention programs, I actively promote cognitive dementia prevention knowledge to older population community members by creating educational handbooks and organizing interactive seminars. This has made me realize that effective health promotion requires solid professional knowledge and community engagement skills.” (P15)*

*“My role has transformed from a passive learner to an active health educator. Through conducting cognitive health education and early screening, I have successfully embraced this new role. I can now independently organize screening activities and feel more confident in my ability to participate with community members in cognitive health promotion activities.” (P17)*

*“I have learned to design personalized cognitive intervention strategies for different older population. For example, I incorporate chess practice into the daily lives of residents who enjoy playing chess, and help residents who like singing join the community choir to maintain their social engagement. This personalized approach is more effective than standardized interventions.” (P5)*

#### 3.3.2 Prevention practice capabilities enhancement

Students could skillfully identify various dementia risk factors and demonstrated sufficient professional confidence and communication skills when consulting with older population about dementia prevention. Students showed increasingly strong professional judgment and implementation abilities in developing personalized dementia prevention plans.

*“Previously, I could only learn about risk factors from textbooks, such as age and genetics. But now I can capture more subtle cues from older population daily behaviors, such as speech hesitation and personality changes, which may indicate cognitive function changes.” (P8)*

*“Now when older population ask me about dementia prevention, I no longer feel nervous. I can explain complex medical concepts in language they understand and provide recommendations based on their specific circumstances.” (P1)*

*“I am increasingly confident in developing different prevention strategies for different older population. For diabetic patients, I focus on blood glucose control; for hypertensive patients, I focus on blood pressure management, and provide specific practical recommendations based on their lifestyles.” (P9)*

#### 3.3.3 Professional identity evolution in prevention

Students have deeply internalized the values of preventive healthcare, forming an integrated medical philosophy that combines prevention and treatment. They recognize that preventive healthcare is an important responsibility for healthcare professionals and have extended this philosophy to personal and family health management. More importantly, students’ career plans have undergone a fundamental transformation, shifting from traditional specialist physician goals toward the ideal of becoming general practitioners with both preventive and therapeutic capabilities.

*“Through my volunteer experience, I discovered that most older population lack awareness of cognitive health and only focus on managing diseases like hypertension, diabetes, and other conditions. This reality gap completely changed my understanding of preventive healthcare.” (P16)*

*“Now I believe that a doctor who only knows how to treat diseases but not prevent them is incomplete, just like a firefighter who only knows how to extinguish fires but not prevent disasters. We should cultivate preventive awareness during our studies and take on the responsibility of being health guardians.” (P7)*

*“I began applying preventive knowledge within my family, teaching my parents and grandparents methods to prevent dementia. I feel that this program’s impact has extended from the community to my family.” (P12)*

*“This program completely changed my career planning. I previously only wanted to be a clinical physician, but now I'm beginning to consider the field of preventive healthcare because I believe prevention is more meaningful than treatment.” (P19)*

### 3.4 Personal experience-driven preventive healthcare awakening

This theme reflects the profound cognitive transformation that medical students undergo through their personal experiences of witnessing family members suffer from illness. When medical students observe their family members enduring the pain and progression of disease, they begin to question the treatment-centered approach emphasized in traditional medical education and develop a deeper recognition of the importance and necessity of preventive healthcare.

#### 3.4.1 Impact of family dementia experience

Medical students experience profound emotional and cognitive impact through direct observation and personal experience of their family members’ illness trajectory. During this period, they not only witness the immense suffering caused by the decline in their family members’ cognitive function, but also experience firsthand the heavy burden of family caregiving. This dual impact prompts them to engage in deep reflection on the limitations of the traditional treatment-focused medical model.

*“When I saw my grandmother gradually forgetting our names and failing to recognize familiar faces, I realized how devastating dementia can be for patients and their families. This made me think: if we could take preventive measures earlier, perhaps we wouldn't have to endure such suffering.” (P5)*

*“Watching my parents care for my grandmother with Alzheimer’s disease while trying to maintain their lives and work, the financial burden and endless worries made me realize that dementia is truly a family disease. From then on, I began thinking about how to help other families avoid such predicaments through prevention.” (P13)*

#### 3.4.2 Experience-triggered prevention awareness

After experiencing their family members’ illnesses, medical students actively sought relevant preventive knowledge and re-examined their future medical practice directions. They underwent a significant transformation from traditional “treating disease” thinking to embracing concepts of “preventing disease” and “promoting health.” This awakening was spontaneous and autonomous, reflecting their shift from passively accepting medical education to actively exploring preventive healthcare approaches.

*“After witnessing my grandmother’s struggle with dementia, I began independently exploring preventive methods. Through our club training, I learned that modifiable factors such as diet, exercise, and social interaction can help prevent or delay cognitive decline. If we had known these strategies earlier, could we have helped my grandmother maintain her cognitive health?” (P18)*

*“My family’s experience with illness completely changed my perspective on the medical profession. I no longer simply want to treat patients; instead, I hope to focus on maintaining health and preventing disease. I believe this approach can have a greater impact on patients and their families.” (P17)*

## 4 Discussion

### 4.1 Principle finding

Based on Mezirow’s TLT, this study revealed that the TL process of medical students participating in community dementia prevention programs manifested through four themes. The first three themes formed a sequential progression that facilitated students’ perspective transformation from the traditional curative-focused medical model toward a more prevention-oriented approach. These themes included: (i) cognitive awakening and reflection; (ii) skill acquisition; and (iii) practice integration and role reconstruction. Additionally, a fourth theme emerged independently, characterized by prevention healthcare-oriented awakening driven by students’ personal experiences and emotional connections with those they served. Through the three TLT-based themes and the additional experiential theme, conceptual transformation regarding preventive healthcare was achieved among medical students.

The cognitive awakening and reflection stage in preventive healthcare transformation involved three key aspects: confronting disorienting dilemmas, critical self-examination, and role cognition transformation. This study found that when medical students confronted situations that challenged their existing cognitive frameworks during community practice, they naturally developed internal motivation for self-assessment and deep reflection to changed their roles. This corresponded with Mezirow’s TLT, which emphasized that disorienting dilemmas represented “the initial stage of TL” (32). Our findings revealed that volunteers re-examined the value of medical practice when confronted with the gap between theoretical knowledge and the complexities of real-world healthcare delivery. This reflection aligned with the theory-practice gap in healthcare education, where students routinely encounter clinical situations that require knowledge and skills inadequately addressed in their formal curricula (33). This process sparked their initial awareness of preventive healthcare’s importance beyond traditional curative approaches, establishing a solid cognitive foundation for subsequent professional identity development. Although our findings aligned with previous evidence on cultivating deep thinking abilities (34) and theory-to-practice integration (35), we provided deeper insights into how community dementia prevention programs specifically trigger professional role transformation. In this study, volunteers participating in community dementia prevention programs reflected on their preventive knowledge deficits and demonstrated transformation in their perception of professional roles. Notably, our study found that this role transformation occurred through direct engagement with community health challenges, thereby expanding beyond the treatment-focused approach to patients commonly emphasized in medical training. This finding was in line with WHO’s social accountability framework and suggested that community-engaged education appeared to have potential for reshaping students’ understanding of their professional role (36).

Following the preliminary awakening phase, students navigated through the stage of “skill acquisition” to resolve challenges. They moved this stage through three progressive processes: knowledge framework construction, intervention strategy development, and practical skill cultivation. Our results revealed that medical students, after encountering low awareness of dementia prevention among older adults, felt a professional obligation to acquire relevant knowledge to address this community health gap. This finding aligned with previous study suggesting that students exposed to underserved populations developed greater commitment to addressing health disparities and were more likely to pursue primary care fields that served community needs (37). Additionally, faced with diverse community resources and varying older population needs, volunteers in our study enhanced their practical problem-solving abilities by developing community-specific intervention activities. This real-world, challenge-driven approach required medical students to identify problems, analyze available resources, and create tailored solutions. These findings were consistent with previous research demonstrating that such a problem-based learning approach was more effective than lecture-based learning in developing problem-solving skills (38).

After the skill acquisition phase, participants entered the final and most transformative stage of their TL process. The practice integration and role reconstruction stage in this study manifested in three key dimensions: health promoter role practice, prevention practice capabilities enhancement, and professional identity evolution in prevention healthcare. In this final stage of TL, volunteers recognized the integrated nature of prevention and treatment, embraced preventive healthcare as a core professional responsibility, applied family-centered disease prevention practices, and repositioned their career plans to incorporate prevention-focused competencies. Our findings showed that participants improved their prevention practice capabilities through community health promotion roles, consistent with Ferreira et al.’s findings (39). This revealed that the TL process occurred through community-based dementia prevention programs, which differed from traditional clinical education approaches that primarily focus on hospital-based training environments (40). Additionally, our study found that practice integration through community dementia prevention programs reconstructed medical students’ professional roles, transforming them from treatment-centered to active health advocates as they became aware of prevention as being integral to their medical identity. This finding in line with previous research demonstrating that community-based experiences could facilitate TL among medical students, particularly in reconstructing professional identity from traditional biomedical models toward more holistic, prevention and advocacy-oriented practice approaches (41). However, professional identity formation is an ongoing and dynamic process (42). Future studies should examine whether community educational interventions produce deep and sustainable changes in professional identity development among medical students. This transformation reflected the progressive nature of TL in practice-based education, where students underwent cognitive awakening and reflection about the broader scope of healthcare, acquired new skills in community engagement and prevention strategies, and ultimately integrated these experiences to reconstruct their professional identity as health advocates rather than solely disease treaters or carers.

Our research revealed a significant new discovery that personal family experiences with dementia emerged as a powerful motivating factor driving medical students’ active engagement in dementia prevention activities. In Theme 4, volunteers who personally experienced the distress and caregiving burden of family members’ cognitive decline developed profound understanding of dementia prevention importance. This experience uniquely motivated them to actively explore preventive knowledge and transition toward prevention-oriented practices, that extended beyond TLT’s scope and highlighted the transformative influence of lived experiences on learning motivation. Although our findings aligned with previous studies showing that students in dementia-related initiatives often had personal family connections (43), we provided deeper insights into how these experiences specifically translate into proactive learning behaviors and career orientation. This corresponded with Self-Determination Theory, which emphasized that autonomous motivation leads to better academic outcomes than controlled motivation driven by external pressures (44). Importantly, our study further revealed that volunteers with family dementia experiences were more likely to pursue career aspirations in preventive healthcare. While this finding was consistent with existing literature supporting meaningful personal experiences as catalysts for professional development (45), our research provided novel evidence within the dementia-specific context.

In our study, personal experiences uniquely motivated volunteers to actively explore preventive knowledge and transition toward prevention-oriented practices, suggesting that lived experiences create a synergistic effect with structured community-based learning. This finding represents a departure from Mezirow’s original TLT framework. The intimate exposure to dementia through family experiences appears to intensify the disorienting dilemma and accelerate the critical reflection processes central to TLT, demonstrating how personal biographical factors can extend transformative learning models in medical education settings and represent a complementary pathway to perspective transformation in health professions education.

### 4.2 Implications for medical education

Firstly, this study demonstrated the significance of TLT in facilitating medical students’ shift from curative-focused to prevention-oriented thinking patterns. Through cognitive awakening, reflection, skill acquisition, and role reconstruction, students developed new perspectives on preventive healthcare while simultaneously building their practice capability and professional identity. Accordingly, medical educators should intentionally design learning environments that incorporate TLT frameworks, encouraging critical reflection and knowledge reconstruction to prepare future healthcare professionals as change agents in evolving healthcare systems. Additionally, we recommend that medical schools integrate community healthcare practice into their core curricula and employ problem-driven learning approaches, thereby establishing pathways that connect theory with practice through structured community opportunities while fostering professional responsibility in addressing health disparities within authentic community settings. Notably, community healthcare practice should be designed to incorporate more situations that trigger disorienting dilemmas, utilizing case discussions and reflective reports to deepen students’ critical self-examination processes. Establishing mentorship is recommended to guide students through effective internal reflection and role cognition transformation when encountering cognitive conflicts.

Based on previous research highlighting the importance of leveraging students’ personal motivations to enhance learning quality (46), our findings suggested several educational implications for medical schools. Firstly, medical schools should systematically incorporate students’ personal experiences as valuable educational resources. This can be achieved through structured experience-sharing sessions and guided reflective activities that stimulated students’ autonomous learning motivation. When students connect their personal experiences with academic content, they may develop stronger intrinsic motivation that lead to deeper and more sustained learning engagement compared to purely theoretical approaches. Secondly, we recommend establishing peer support groups where students with relevant family experiences with a certain health problem can facilitate discussions and share insights with their peers. Such peer-led initiatives can promote collective learning and emotional engagement, creating a supportive learning environment that enhances understanding of the importance of prevention healthcare delivery. Thirdly, for students without direct personal experiences with dementia, educators should develop alternative strategies to create similar emotional connections. These may include patient story narratives, simulation exercises, or community engagement activities that help students develop empathy and authentic understanding of the impact of diseases on patients and families.

### 4.3 Transferability and generalizability of findings

While this study specifically examined dementia prevention education, the TL mechanisms identified offer significant potential for broader application across public health domains. The four-stage transformation process documented in our study provides a generalizable pedagogical framework that extends beyond dementia-specific contexts. This aligns with emerging evidence that TL mechanisms can be successfully adapted across diverse health professional education settings (47). This framework could be effectively adapted to other community-based prevention initiatives. For instance, cardiovascular disease prevention programs could utilize similar community engagement strategies to help students transform their understanding from acute care interventions to long-term lifestyle modification approaches. Cancer screening education could employ comparable mechanisms to shift student perspectives from late-stage treatment to early detection and prevention. Future research should investigate how these TL principles operate across different public health settings and whether the four-stage process we identified manifests similarly in other prevention education contexts.

Even though 21 participants from a single medical university in Guizhou Province, which warrants careful consideration of representativeness and transferability. There are several factors support the broader applicability of our findings. First, Guizhou Province exhibits sociodemographic characteristics common to many developing regions globally, where is economically less developed with below-average education levels and a rapidly aging population. Therefore, our findings may resonate with regions experiencing comparable demographic transitions and healthcare system constraints, both within China and in other middle-income countries. Second, the participants were trained in conventional medical education systems, where curricula traditionally emphasize disease diagnosis and treatment while providing limited preventive healthcare content. The identified perspective shift from curative to preventive orientations through community-based dementia programs is therefore likely transferable to other medical institutions with similar educational models. Third, the TLT framework employed in this study is well-established internationally, and the mechanisms of perspective transformation identified here may resonate with health professional education in diverse cultural contexts (25), particularly in regions experiencing demographic aging and seeking to reorient healthcare systems toward prevention.

## 5 Limitations

Although this study provides valuable insights into understanding the transformation of medical students’ preventive healthcare concepts through community dementia programs, several limitations remain. All participants were from the same medical school and geographic region, which may limit the generalizability of the study. However, this study primarily interviewed second-and third-year medical students who possessed professional knowledge and practical skills in dementia prevention, providing rich information for the research questions. Nevertheless, this also resulted in the neglect of first-year students with insufficient preliminary awareness transformation. Given these limitations, future research should consider studies in different regions and educational contexts, and expand the inclusion scope to all grade levels to better generalize the research findings to all medical students.

## 6 Conclusion

This qualitative study, grounded in Mezirow’s TLT, identified four distinct themes that characterized medical students’ TL journey within community dementia prevention initiatives. Three interconnected themes demonstrated a progressive developmental pathway that enabled students to shift from conventional treatment-centered medical paradigms toward prevention-focused healthcare approaches. These themes included cognitive awakening and reflection, skill acquisition, and practice integration and role reconstruction. A fourth theme emerged as a parallel pathway, representing prevention-oriented consciousness that arose from students’ direct personal encounters and affective bonds with populations they served. The synergistic interaction between the structured TLT-based progression and the personal experiential pathway facilitated a fundamental shift in how medical students conceptualized preventive healthcare. Our study recommends that medical educators should intentionally design learning environments that incorporate TLT frameworks to encourage critical reflection and prepare future healthcare professionals as change agents. Additionally, medical schools are suggested to integrate community healthcare practice into core curricula through problem-driven learning approaches, creating structured pathways that connect theory with practice to foster professional responsibility. Community-based learning should incorporate disorienting dilemmas through case discussions and reflective reports, supported by mentorship to guide students through cognitive conflicts and role transformation. Furthermore, medical schools should systematically leverage students’ personal experiences as educational resources through experience-sharing sessions and peer support groups.

## Data Availability

The original contributions presented in the study are included in the article/Supplementary material, further inquiries can be directed to the corresponding author/s.
