# Supplementary material for: The curative-to-preventive perspective shift of medical students through community dementia programs: a qualitative study based on transformative learning theory
Source: Front Public Health. 2026 Jan 12;13:1708455. doi: 10.3389/fpubh.2025.1708455 (PMC12832732; doi:10.3389/fpubh.2025.1708455)
Supplement: Supplementary file 1 [file Table_1.DOCX]

Supplementary Material

**Supplementary Table 1**：The overview of themes, sub-themes, categories and meaning unit

| **Themes** | **Sub-themes** | **Categories** | **Meaning Unit** |
| --- | --- | --- | --- |
| Cognitive awakening and reflection | Encountering disorienting dilemmas | Gap between theoretical knowledge and community healthcare needs | “Our curriculum primarily focused on the pathogenesis, symptoms, and care of dementia, with little mention of prevention strategies. But when I began working in the field, I realized what really needed to happen was to help seniors develop preventative awareness and guide them toward a healthier lifestyle.”  “I used to think that medicine was all about diagnosing and treating diseases effectively. However, my recent community experience with dementia prevention has taught me that prevention is just as important as treatment. While we know how to fight diseases, we often don’t know much about how to actually promote health. At first, when older adults in the community asked me ‘What can I do to prevent dementia?’ I found it really challenging to give them practical advice beyond what I’d learned from textbooks.” |
|  |  | Curriculum gaps in preventive healthcare education | “In our medical school, we have curriculums like pathology, pharmacology, and diagnostics, but there are almost no specialized courses teaching us how to actually implement health promotion and disease prevention.”  “When conducting dementia prevention activities in the community, I realized that we needed to consider not only the accuracy of medical content but also how to enhance healthy behaviors, address cultural differences, and address the various concerns and worries of seniors about dementia prevention. These practical skills are almost completely missing from our current medical education.”  “Our medical education rarely prepares us for actual community work. When I first went into the community to talk with elderly residents, I felt totally lost—I had no idea how to communicate effectively or build trust with them. It really made me realize how much our training focuses on clinical treatment while completely ignoring the skills we actually need for preventive healthcare in community settings.”  “What we learn in class is all about disease mechanisms and approaches to caring for patients, but there’s hardly any content on how to deliver health education for disease prevention in the community. Through this project, I found that medical students really lack knowledge and skills in preventive healthcare. I honestly think our school should add more courses or training in this area.” |
|  |  | Recognition of current medical education limitations | “I used to think that medical professionals’ responsibilities were solely to treat patients. But when I saw that the mental health of the elderly in our community was significantly better than that of those already suffering from dementia, I suddenly realized: Wouldn’t it be more meaningful to help them maintain this state of mind?”  “I used to think that healthcare providers’ professional value could only be demonstrated in hospital wards and operating rooms. But now I’ve realized that teaching cognitive training to elderly people in the community and guiding them on proper nutrition—these seemingly simple activities actually have much more long-term significance.”  “Looking back, I think our medical curriculum has a fundamental limitation-it teaches us to be reactive rather than proactive. We learn to wait for diseases to develop and then treat them, but we’re not really trained to think about preventing them from occurring. This community experience showed me how much our education emphasizes cure over prevention.” |
|  | Self-examination and critical reflection | Prevention knowledge deficits | “Previously, I knew almost nothing about dementia prevention and had no understanding of how to prevent it or control risk factors. After the training, I realized that many daily behaviors could serve a preventive role.”  “When I first started learning about prevention knowledge, I realized my knowledge was pretty limited. For example, stuff like how nutritional balance affects brain health, or the importance of social activities-these topics are barely touched on in our regular courses. I’ve come to realize that there’s still so much I need to learn.” |
|  |  | Insufficient basic knowledge | “I used to think dementia was just about memory loss, but it turns out I had such a narrow view of it. There are also issues with executive function, language deterioration, and a bunch of other stuff I had no clue about before. This really made me realize how many gaps I still have in my basic knowledge.”  “The dementia screening made me realize how little I knew. Although I had learned about MMSE and MoCA in class, I felt overwhelmed when applying them in practice. This made me realize the huge gap between rote memorization and actual application.” |
|  |  | Lack of practical application knowledge | “I realized there was a huge gap between textbook knowledge and actual practice. We had learned multidisciplinary prevention theories, but I didn’t know how to apply these concepts to the specific situations of elderly individuals. Because each person’s situation was different, it was difficult for me to develop practical plans that truly suited them.”  “The biggest challenge for me was helping older people stick to prevention measures. I can explain the importance of regular physical activity and balanced diets, but when it comes to creating feasible, personalized plans based on each person’s specific circumstances—that’s where I realized I lack the practical skills. There existed a gap between the theoretical knowledge we have and the hands-on experience needed to work effectively with real patients.” |
|  | Role perception transformation | Identity shift from healer to prevention advocate | “Before participating in this dementia prevention plans, I thought healthcare meant treating patients in hospitals. But working with community members made me realize that we can actually help prevent diseases from occurring in the first place.”  “It’s been eye-opening to see how we can make a real difference by educating people about dementia prevention rather than just waiting to treat it later. This experience has really changed how I see my future role as a healthcare professional—from someone who just fixes problems to someone who helps prevent them.”  “Working on this project really shifted my perspective. Instead of just learning how to treat diseases, I started seeing myself as someone who could actually protect people’s health before they got sick. It made me think—why wait until someone has dementia to help them? Why not teach them how to prevent it now? This whole experience made me realize that being a nurse isn’t just about caring and healing–it’s about keeping people healthy in the first place. That feels way more meaningful to me.” |
|  |  | Recognition of preventive healthcare values | “I used to think that prevention was not as important as treatment. But through my involvement in the prevention programs, I realized that preventing cognitive decline is more valuable.”  “To be honest, I used to think preventive medicine was kind of boring compared to emergency medicine or surgery. But this project taught me that dementia can’t be cured, only prevented-and that completely shifted my perspective. Now I actually see prevention as this powerful tool that can transform entire communities, especially when treatment options are so limited.” |
| Skill acquisition | Building prevention knowledge framework | Learning to raise awareness | “I have found that elderly people have misconceptions about dementia and lack basic prevention knowledge. Therefore, raising awareness of prevention among the elderly is crucial. Only by helping them understand the importance of prevention can they truly participate in the program.”  “The health promotion activities we held in the community made the elderly realize that dementia can be prevented, which changed their perception. I also deeply understand that public health education can play an important role in disease prevention.”  “When I first joined this project, I found that many older adults had no concept of dementia prevention at all. They thought ‘poor memory in old age is normal. ’I realized that we first need to change their mindset and let them know that prevention is useful and necessary. This is more important than simply teaching prevention methods.” |
|  |  | Identifying optimal prevention timing | “Through this project, I discovered there’s a critical golden period for dementia prevention, particularly during mild cognitive impairment. Early detection and intervention can significantly slow disease progression. Using MMSE, MoCA and other cognitive tools to screen community older adults, we found many had subtle memory issues that neither they nor their families recognized.”  “Learning to use cognitive screening tools taught me that early screening isn’t just about detecting existing problems—it’s about identifying high-risk individuals whose cognitive function is declining but hasn’t yet met dementia diagnostic criteria.”  “During the community screening process, I discovered that many elderly people ignore slight memory changes, considering them normal signs of aging. However, when we emphasize the importance of early screening and inform them that intervention during the mild cognitive decline stage helps maintain better quality of life, they all actively participate.” |
|  |  | Mastering approaches to prevent dementia | “Through training, I learned that risk factors for cognitive impairment include hypertension, diabetes, hyperlipidemi, and others. During health education, we should teach elderly people to control these chronic diseases through diet, exercise, medication, and other methods to reduce the risk of developing the condition.”  “I have learned to develop personalized dementia prevention intervention plans for different elderly people, incorporating various methods such as cognitive training, aerobic exercise, social activities, and the Mediterranean diet. The most important thing is to tailor the intervention strategies to each person’s physical condition, interests, and living environment.”  “Through systematic learning, I have mastered many prevention methods that I didn’t know before, such as the Mediterranean diet, Tai Chi, and puzzle games. These seemingly simple activities actually have a scientific basis. I have come to understand that preventing dementia does not rely on a single method, but requires comprehensive lifestyle interventions.” |
|  | Community intervention design | Intervention design considering community conditions | “Considering the community’s resource constraints, we used to play cards and household items to design simple memory games, integrating cognitive training with daily activities. This approach provided effective dementia prevention interventions without requiring expensive resources.”  “Due to limited space in the community activity rooms, it was difficult for all elderly participants to sit down, resulting in low participation. To solve this, we developed a nine-square grid exercise using square dance music that they enjoy. This allowed everyone to stand, move, and engage in cognitive activities within the small space. The familiar music motivated them to participate, while the grid format provided effective cognitive stimulation, addressing both space constraints and their interests.”  “We found that every community has different conditions. Some communities have better facilities, while others are quite basic. So, we can’t copy standard plans from textbooks; we must adapt to local conditions. For example, in communities without multimedia equipment, we created simple visual aids like colorful posters showing brain-healthy foods and used real fruits and vegetables as props during our nutrition education sessions. Older adults could actually touch the foods while we explained their benefits for brain health, which made the learning experience more engaging and memorable.” |
|  |  | Converting prevention theories into practical approaches | “We learned to simplify professional intervention methods into forms that elderly people could easily accept. For example, when using the MMSE scale, we changed ‘100 minus 7’ to ‘If you have 100 yuan and spend 7 yuan on groceries, how much money do you have left?’ This approach was more closely related to their daily life experiences.”  “Previously, our attention training required the elderly to stare at a computer screen and perform reaction exercise, but we found that most of them had difficulty operating a computer. Therefore, we changed it to a paper-based game called ‘Find the Differences’, where they are given two similar pictures and asked to find five differences. We found that this method is more suitable for exercising their cognitive functions.”  “Through this experience, I developed skills in translating complex cognitive training methodologies into accessible formats for elderly participants. For example, we transformed computer-based memory training into interactive ‘story retelling’ activities, and converted executive function assessments into ‘simulated shopping’ scenario exercises. This approach made the interventions both engaging and practically relevant to their daily lives.” |
|  |  | Tailored prevention plan development skills | “During the implementation of early intervention, I learned from the literature and our supervisors that a single intervention method has limited effects. Following evidence-based guidelines, I realized that only by adopting a comprehensive multi-dimensional intervention can the best results be achieved. For instance, while organizing the elderly to participate in Tai Chi exercises, we also teach them to play card games to train their cognitive functions and encourage them to take part in community activities to enhance social interaction.”  “Each elderly person’s situation was different-some preferred physical activities while others preferred sedentary activities, and their educational levels also varied. We had to design personalized prevention plans based on each individual’s characteristics, which tested our professional competency and innovative thinking.”  “When developing personalized plans, I learned to comprehensively consider multiple factors such as the elderly’s physical condition, interests, and family support. For example, for elderly with arthritis, we recommend finger exercises instead of Tai Chi; for elderly living alone, we emphasize more the importance of social activities. This personalized approach makes elderly more willing to cooperate.” |
|  | Development of practical skills | Professional terminology conversion | “I learned how to use simple and understandable expressions to help elderly participants understand professional medical terms, such as explaining “verbal fluency difficulty” with “having the words on the tip of your tongue but not being able to get them out.” This approach made me realize that bridging the gap between medical jargon and everyday language was crucial for successful health education in the community.”  “For me, it is quite challenging to explain cognitive dysfunction to the elderly. So, I have learned to use the concept of memory instead. For instance, I would say that if one gets this disease, the main issue is that their memory would be worse than that of their peers. If no intervention is made, it might affect their daily lives.”  “Seeing their bewildered expressions, I knew my explanation had exceeded their comprehension. I would immediately change my approach, using familiar life scenarios to help them understand. For example, when explaining executive function, I would say it’s like cooking-you need to remember the sequence of washing vegetables, cutting vegetables, and cooking vegetables.” |
|  |  | Mastering health education methods | “Incorporating fun activities making older adults love to join our health education session! I’ve learned to design interesting games that benefit cognitive function to allow elderly participate our health education activities actively. So, I feel that health education isn’t just one-way information delivery, it’s about creating two-way effective interactive communication.”  “I realized that the foundation of health education lies in changing behavior, not merely transmitting information. Therefore, we focus on helping them find their own motivation and practical methods for change. For example, when promoting executive function for dementia prevention, we share the power of daily puzzles and help participants set achievable goals.” |
|  |  | Learning to assess community residents’ needs | “Through field research experience, I discovered that different communities and population groups have vastly different needs. Elderly residents in communities with higher education levels tend to place greater emphasis on scientific evidence, while elderly people living alone require additional emotional support and social connections. This made me understand the importance of needs assessment.”  “At first, we designed activities based on what we thought was helpful, but this proved ineffective. By using surveys, interviews, and observations, I learned to understand residents’ real needs. I have realized that they weren’t interested in complex medical theories. Instead, they cared more about practical things, like what daily habits could help keep their memory sharp and how to stay independent for as long as possible. This completely changed my approach to community education on dementia prevention.” |
|  |  | Developing patience and empathy with older adults | “This dementia prevention project taught me the importance of communication skills. Working with elderly participants, I realized that effective health education requires patience to explain procedures clearly and empathy to address their fears about memory loss. These skills turned out to be essential tools in healthcare practice.”  “When I first communicated with elderly people, I was always anxious, hoping they could quickly understand what I meant. But gradually I found that elderly people need more time to accept new information, and they also have their own life experiences and established perspectives. I learned to slow down my speech, have more patience, and listen more attentively, which actually made communication much more effective.”  “Through long-term contact, I began to understand the psychological state of elderly people. They feel anxious about the decline in their physical functions and confused about the transformation of their social roles. After understanding them, I became more empathetic in communication and was able to think from their perspective.” |
| Practice integration and role reconstruction | Health promoter role practice | Active dissemination of dementia prevention knowledge | “Through the cognitive impairment prevention programs, I actively promote cognitive dementia prevention knowledge to elderly community members by creating educational handbooks and organizing interactive seminars. This has made me realize that effective health promotion requires solid professional knowledge and community engagement skills.”  “Taking on the role of prevention advocate completely transformed how I understand healthcare delivery. Organizing community workshops on cognitive health taught me that effective health promotion isn’t just about sharing medical facts—it’s about making complex information accessible and culturally relevant to different audiences.” |
|  |  | Capable of independently conducting dementia early screening activities | “My role has transformed from a passive learner to an active health educator. Through conducting cognitive health education and early screening, I have successfully embraced this new role. I can now independently organize screening activities and feel more confident in my ability to participate with community members in cognitive health promotion activities.”  “When I first started doing community screenings, I honestly felt pretty lost. Talking to older adults was intimidating-they have so much life experience, and here I am, just a student trying to help them. But as I kept participating in the project’s training sessions, I started to understand how to communicate better. By the end of the program, I was able to run screening sessions on my own and even helped train newer student volunteers.” |
|  |  | Learning to guide residents in early cognitive intervention | “What really changed for me was understanding how preventive healthcare actually works in practice. Before this project, I thought prevention just meant telling people to exercise and eat well. But working with the elderly community taught me that effective prevention requires understanding their daily routines, cultural backgrounds, and individual barriers.”  “I have learned to design personalized cognitive intervention strategies for different elderly residents. For example, I incorporate chess practice into the daily lives of residents who enjoy playing chess, and help residents who like singing join the community choir to maintain their social engagement. This personalized approach is more effective than standardized interventions.” |
|  | Prevention practice capabilities enhancement | Skillfully identify risk factors for dementia | “I’ve learned to identify early warning signs that textbooks don’t really cover-like subtle changes in social behavior or daily functioning that might indicate cognitive decline.”  “Previously, I could only learn about risk factors from textbooks, such as age and genetics. But now I can capture more subtle cues from elderly people’s daily behaviors, such as speech hesitation and personality changes, which may indicate cognitive function changes.”  “Through community work, I’ve gradually learned to observe elderly people’s living habits and spot factors that might affect cognitive health, such as social isolation, lack of exercise, and poor dietary habits. This kind of hands-on learning is so much more impactful than classroom learning.” |
|  |  | More confident in answering residents’ consultations about dementia prevention | “I can now confidently answer residents’ various questions about dementia prevention—whether they are asking about brain-healthy foods, cognitive exercises, or when to seek professional screening. I feel comfortable explaining complex prevention concepts in simple terms and can provide personalized recommendations based on their individual situations.”  “Now I can sit down with an elderly person and clearly explain things like risk factors for dementia, memory exercises, and lifestyle modifications. I feel like I’ve grown from being just a student who memorizes textbooks to someone who can actually help people in real life.”  “Now when elderly people ask me about dementia prevention, I no longer feel nervous. I can explain complex medical concepts in language they understand and provide recommendations based on their specific circumstances.” |
|  |  | Increasingly confident in developing personalized dementia prevention plans | “Now I can develop comprehensive prevention strategies that actually fit into people’s real lives, not just theoretical recommendations.”  “When I first started making prevention plans, I would just copy suggestions from textbooks. Now I develop truly suitable personalized plans based on each elderly person’s specific situation, such as their interests, physical condition, and family support.”  “I am increasingly confident in developing different prevention strategies for different elderly individuals. For diabetic patients, I focus on blood glucose control; for hypertensive patients, I focus on blood pressure management, and provide specific practical recommendations based on their lifestyles.” |
|  | Professional identity evolution in prevention | Recognition of prevention and treatment as integrated | “Through my volunteer experience, I discovered that most elderly people lack awareness of cognitive health and only focus on managing diseases like hypertension, diabetes, and other conditions. This reality gap completely changed my understanding of preventive healthcare.”  “I used to think prevention and treatment were two separate things, and doctors mainly treat diseases. But after working in the community, I realized that prevention is actually an extension of treatment, and early prevention is more important and effective than later treatment.”  “I found that prevention and treatment are actually a continuous process without clear boundaries. For example, for an elderly person with mild cognitive impairment, we need to both ‘treat’ their existing problems and ‘prevent’ them from developing dementia. This made me realize that true medicine should be whole-process medicine, requiring healthcare providers to be involved from when people are healthy all the way to when they get sick.” |
|  |  | Recognizing disease prevention as an important responsibility of healthcare providers | “Through this dementia prevention project, I came to recognize that disease prevention is not optional for healthcare providers-it’s a core professional responsibility. I used to think doctors and nurses were mainly responsible for treating sick patients, but now I believe we have an ethical obligation to proactively protect community health and prevent diseases before they occur. This shift in understanding has made me realize that preventive healthcare should be every medical professional’s duty, just as important as clinical treatment.”  “I began to think about the social role of doctors differently. We shouldn’t just sit in hospitals waiting for patients to come to us, but should actively go into communities to help people prevent diseases. This proactive prevention work should become a basic professional competency for every healthcare provider, as important as being able to diagnose and prescribe medications.”  “Now I believe that a doctor who only knows how to treat diseases but not prevent them is incomplete, just like a firefighter who only knows how to extinguish fires but not prevent disasters. We should cultivate preventive awareness during our studies and take on the responsibility of being health guardians.” |
|  |  | Family chronic disease prevention practice | “I began applying preventive knowledge within my family, teaching my parents and grandparents methods to prevent dementia. I feel that this program’s impact has extended from the community to my family.”  “Now I actively pay attention to my family’s health status. I no longer just ask ‘how are you feeling’ like before, but observe changes in their cognitive function, blood pressure and blood sugar level. I would also remind them to pay attention to keep good lifestyle to prevent this chronic disease. This role change makes me feel like I’m really becoming a healthcare professional.”  “Through practicing prevention knowledge at home, I started implementing the dementia prevention strategies I learned with my own family members. I helped my parents establish regular cognitive exercise routines, encouraged my grandparents to adopt brain-healthy dietary habits like the Mediterranean diet, and taught them simple memory games. Seeing my family actively engage in these preventive measures and witnessing their improved awareness of cognitive health made me realize how prevention knowledge can truly transform families when properly applied in daily life.” |
|  |  | Career aspirations in preventive healthcare | “This program completely changed my career planning. I previously only wanted to be a clinical physician, but now I’m beginning to consider the field of preventive healthcare because I believe prevention is more meaningful than treatment.”  “I began to re-examine the value of different medical specialties. I used to think surgeons were the most impressive because they could perform operations to save lives; now I think doctors engaged in prevention work are also remarkable because they can help more people avoid needing surgery. Maybe my future doesn’t necessarily have to be on the operating table; doing prevention work in communities is equally meaningful.”  “Now my ideal is no longer just to become an excellent clinical doctor, but to become a general practitioner who can both treat and prevent diseases. I think this is a career choice that truly aligns with the development trend of modern medicine.” |
| Personal experience-driven preventive healthcare awakening | Impact of family dementia experience | Witnessing the suffering caused by family members’ cognitive decline | “When I saw my grandmother gradually forgetting our names and failing to recognize familiar faces, I realized how devastating dementia can be for patients and their families. This made me think: if we could take preventive measures earlier, perhaps we wouldn’t have to endure such suffering.”  “My grandfather had always been the wisest person in our family, but dementia stripped him of his dignity and independence. Watching him struggle with the simplest daily tasks, which he once performed with ease, made me truly realize the critical importance of protecting cognitive health.” |
|  |  | Experiencing the heavy burden of family caregiving | “Taking care of my grandmother with dementia has been emotionally and physically exhausting for our entire family. My parents took turns staying with her 24/7, and I saw how this disease doesn’t just affect the patient—it changes everyone’s life. This experience made me realize how much suffering could be prevented if we focused more on early intervention.”  “Watching my parents care for my grandmother with Alzheimer’s disease while trying to maintain their lives and work, the financial burden and endless worries made me realize that dementia is truly a family disease. From then on, I began thinking about how to help other families avoid such predicaments through prevention.” |
|  | Experience-triggered prevention awareness | Active exploration of prevention knowledge | “After my grandmother showed signs of cognitive decline during routine screening, I started reading everything I could find about dementia prevention. I wanted to understand if there were evidence-based interventions that could slow down her cognitive deterioration or prevent it from progressing to dementia. This self-directed learning made me realize how much preventive knowledge exists that we are not taught in medical school, and how important it is for future doctors to understand early intervention and prevention strategies.”  “After witnessing my grandmother’s struggle with dementia, I began independently exploring preventive methods. Through our club training, I learned that modifiable factors such as diet, exercise, and social interaction can help prevent or delay cognitive decline. If we had known these strategies earlier, could we have helped my grandmother maintain her cognitive health?” |
|  |  | Reorientation toward prevention-focused medical practice | “My family’s experience with illness completely changed my perspective on the medical profession. I no longer simply want to treat patients; instead, I hope to focus on maintaining health and preventing disease. I believe this approach can have a greater impact on patients and their families.”  “Seeing the devastating effects of dementia on my own family made me realize that preventive medicine should be every doctor’s responsibility. I now plan to integrate prevention strategies into whichever specialty I choose because I don’t want other families to go through what mine did. Early intervention and prevention education could prevent so much suffering.” |

**Supplementary Table 2**：The overview of themes, sub-themes, and illustrative quotes

| **Themes** | **Sub-themes** | **Illustrative quotes** |
| --- | --- | --- |
| Cognitive awakening and reflection | Encountering disorienting dilemmas | “Our curriculum primarily focused on the pathogenesis, symptoms, and care of dementia, with little mention of prevention strategies. But when I began working in the field, I realized what really needed to happen was to help seniors develop preventative awareness and guide them toward a healthier lifestyle.”  “When conducting dementia prevention activities in the community, I realized that we needed to consider not only the accuracy of medical content but also how to enhance healthy behaviors, address cultural differences, and address the various concerns and worries of seniors about dementia prevention. These practical skills are almost completely missing from our current medical education.”  “I used to think that medical professionals’ responsibilities were solely to treat patients. But when I saw that the mental health of the elderly in our community was significantly better than that of those already suffering from dementia, I suddenly realized: Wouldn’t it be more meaningful to help them maintain this state of mind?” |
|  | Self-examination and critical  reflection | “Previously, I knew almost nothing about dementia prevention and had no understanding of how to prevent it or control risk factors. After the training, I realized that many daily behaviors could serve a preventive role.”  “The dementia screening made me realize how little I knew. Although I had learned about MMSE and MoCA in class, I felt overwhelmed when applying them in practice. This made me realize the huge gap between rote memorization and actual application.”  “I realized there was a huge gap between textbook knowledge and actual practice. We had learned multidisciplinary prevention theories, but I didn’t know how to apply these concepts to the specific situations of elderly individuals. Because each person’s situation was different, it was difficult for me to develop practical plans that truly suited them.” |
|  | Role perception transformation | “Before participating in this dementia prevention plans, I thought healthcare meant treating patients in hospitals. But working with community members made me realize that we can actually help prevent diseases from occurring in the first place.”  “I used to think that prevention was not as important as treatment. But through my involvement in the prevention programs, I realized that preventing cognitive decline is more valuable.” |
| Skill acquisition | Building prevention knowledge framework | “I have found that elderly people have misconceptions about dementia and lack basic prevention knowledge. Therefore, raising awareness of prevention among the elderly is crucial. Only by helping them understand the importance of prevention can they truly participate in the program.”  “During the community screening process, I discovered that many elderly people ignore slight memory changes, considering them normal signs of aging. However, when we emphasize the importance of early screening and inform them that intervention during the mild cognitive decline stage helps maintain better quality of life, they all actively participate.”  “Through training, I learned that risk factors for cognitive impairment include hypertension, diabetes, hyperlipidemia, and others. During health education, we should teach elderly people to control these chronic diseases through diet, exercise, medication, and other methods to reduce the risk of developing the condition.” |
|  | Community intervention design | “Considering the community’s resource constraints, we used playing cards and household items to design simple memory games, integrating cognitive training with daily activities. This approach provided effective dementia prevention interventions without requiring expensive resources.”  “We learned to simplify professional intervention methods into forms that elderly people could easily accept. For example, when using the MMSE scale, we changed ‘100 minus 7’ to ‘If you have 100 yuan and spend 7 yuan on groceries, how much money do you have left?’ This approach was more closely related to their daily life experiences.”  “Each elderly person’s situation was different-some preferred physical activities while others preferred sedentary activities, and their educational levels also varied. We had to design personalized prevention plans based on each individual’s characteristics, which tested our professional competency and innovative thinking.” |
|  | Development of practical  skills | “Seeing their bewildered expressions, I knew my explanation had exceeded their comprehension. I would immediately change my approach, using familiar life scenarios to help them understand. For example, when explaining ‘executive function’, ‘I would say,’ It’s like cooking-you need to remember the sequence of washing vegetables, cutting vegetables, and cooking vegetables.”  “I realized that the foundation of health education lies in changing behavior, not merely transmitting information. Therefore, we focus on helping them find their own motivation and practical methods for change. For example, when promoting executive function for dementia prevention, we share the power of daily puzzles and help participants set achievable goals.”  “Through field research experience, I discovered that different communities and population groups have vastly different needs. Elderly residents in communities with higher education levels tend to place greater emphasis on scientific evidence, while elderly people living alone require additional emotional support and social connections. This made me understand the importance of needs assessment.”  “Through long-term contact, I began to understand the psychological state of elderly people. They feel anxious about the decline in their physical functions and confused about the transformation of their social roles. After understanding them, I became more empathetic in communication and was able to think from their perspective.” |
| Practice integration and role reconstruction | Health promoter  role  practice | “Through the cognitive impairment prevention programs, I actively promote cognitive dementia prevention knowledge to elderly community members by creating educational handbooks and organizing interactive seminars. This has made me realize that effective health promotion requires solid professional knowledge and community engagement skills.”  “My role has transformed from a passive learner to an active health educator. Through conducting cognitive health education and early screening, I have successfully embraced this new role. I can now independently organize screening activities and feel more confident in my ability to participate with community members in cognitive health promotion activities.”  “I have learned to design personalized cognitive intervention strategies for different elderly residents. For example, I incorporate chess practice into the daily lives of residents who enjoy playing chess, and help residents who like singing join the community choir to maintain their social engagement. This personalized approach is more effective than standardized interventions.” |
|  | Prevention practice capabilities enhancement | “Previously, I could only learn about risk factors from textbooks, such as age and genetics. But now I can capture more subtle cues from elderly people’s daily behaviors, such as speech hesitation and personality changes, which may indicate cognitive function changes.”  “Now when elderly people ask me about dementia prevention, I no longer feel nervous. I can explain complex medical concepts in language they understand and provide recommendations based on their specific circumstances.”  “I am increasingly confident in developing different prevention strategies for different elderly individuals. For diabetic patients, I focus on blood glucose control; for hypertensive patients, I focus on blood pressure management, and provide specific practical recommendations based on their lifestyles.” |
|  | Professional identity evolution in  prevention | “Through my volunteer experience, I discovered that most elderly people lack awareness of cognitive health and only focus on managing diseases like hypertension, diabetes, and other conditions. This reality gap completely changed my understanding of preventive healthcare.”  “Now I believe that a doctor who only knows how to treat diseases but not prevent them is incomplete, just like a firefighter who only knows how to extinguish fires but not prevent disasters. We should cultivate preventive awareness during our studies and take on the responsibility of being health guardians.”  “I began applying preventive knowledge within my family, teaching my parents and grandparents methods to prevent dementia. I feel that this program’s impact has extended from the community to my family.”  “This program completely changed my career planning. I previously only wanted to be a clinical physician, but now I’m beginning to consider the field of preventive healthcare because I believe prevention is more meaningful than treatment.” |
| Personal experience-driven preventive healthcare awakening | Impact of  family dementia experience | “When I saw my grandmother gradually forgetting our names and failing to recognize familiar faces, I realized how devastating dementia can be for patients and their families. This made me think: if we could take preventive measures earlier, perhaps we wouldn’t have to endure such suffering.”  “Watching my parents care for my grandmother with Alzheimer’s disease while trying to maintain their lives and work, the financial burden and endless worries made me realize that dementia is truly a family disease. From then on, I began thinking about how to help other families avoid such predicaments through prevention.” |
|  | Experience-  triggered prevention awareness | “After witnessing my grandmother’s struggle with dementia, I began independently exploring preventive methods. Through our club training, I learned that modifiable factors such as diet, exercise, and social interaction can help prevent or delay cognitive decline. If we had known these strategies earlier, could we have helped my grandmother maintain her cognitive health?”  “My family’s experience with illness completely changed my perspective on the medical profession. I no longer simply want to treat patients; instead, I hope to focus on maintaining health and preventing disease. I believe this approach can have a greater impact on patients and their families.” |
